# Intratumoral Corynebacterium is associated with phospholipid metabolic reprogramming and spread through air spaces in lung adenocarcinoma

**DOI:** 10.3389/fcimb.2026.1853739

**Published:** 2026-08-05

**Authors:** Chu Qin, Huachi Jiang, Xiaodong Fan, Haoda Yu, Liang Zhang

**Affiliations:** 1 National Engineering Research Center of Cereal Fermentation and Food Biomanufacturing, School of Biotechnology, Jiangnan University, Wuxi, China; 2 Department of Respiratory Medicine, The Affiliated Wuxi People’s Hospital of Nanjing Medical University, Wuxi, China; 3 Department of Thoracic Surgery, The Affiliated Wuxi People’s Hospital of Nanjing Medical University, Wuxi, China; 4 Engineering Research Center of the Ministry of Education for Wound Repair Technology, Jiangnan University, Affiliated Hospital of Jiangnan University, Wuxi, China

**Keywords:** ABCA3, Corynebacterium, intratumoral microbiome, lung adenocarcinoma, phospholipid metabolism, STAS

## Abstract

**Background:**

Spread through air spaces (STAS) is an adverse invasive pattern in lung adenocarcinoma (LUAD), but reliable biomarkers for preoperative or intraoperative prediction remain limited. We investigated whether the intratumoral microbiome and metabolome are associated with STAS.

**Methods:**

Fresh tumor and paired adjacent non-tumor lung tissues from resected LUAD were analyzed using 5-region 16S rRNA gene sequencing and untargeted metabolomics. Microbiome–metabolome correlations were assessed by Spearman analysis, and receiver operating characteristic curves were used to evaluate diagnostic performance. The clinical relevance of ABCA3 was further explored using GEPIA2. ABCA3 and MMP9 expression was additionally assessed in selected same-cohort tumor samples by qRT-PCR.

**Results:**

Tumor tissues displayed a distinct microbial composition characterized by Corynebacterium enrichment and a metabolomic profile marked by phospholipid remodeling. Corynebacterium abundance was positively correlated with phosphatidylcholine and sphingomyelin species, but negatively correlated with several amino acids. STAS-positive tumors showed higher Corynebacterium abundance and increased membrane-lipid accumulation than STAS-negative tumors. An integrated model combining Corynebacterium, PC(39:6), and SM(d18:1/17:0) showed high apparent discrimination in this exploratory cohort(AUC = 0.98), although external validation is required. In external transcriptomic analyses, higher ABCA3 expression was associated with better overall survival and was inversely correlated with MMP9 expression in LUAD. In selected same-cohort tumor samples, qRT-PCR showed significantly lower ABCA3 expression and higher MMP9 expression in the STAS-positive/Corynebacterium-enriched group than in the STAS-negative/Corynebacterium-low group.

**Conclusions:**

Intratumoral Corynebacterium is associated with phospholipid metabolic reprogramming and a STAS-positive phenotype in LUAD. A combined microbial–metabolic signature may aid STAS risk stratification and suggests a potential association with ABCA3-related lipid homeostasis, but causal relationships require functional validation. Same-cohort qRT-PCR further supported an ABCA3-low/MMP9-high invasive expression pattern in the selected STAS-positive/Corynebacterium-enriched samples.

## 1 Introduction

Lung adenocarcinoma (LUAD) remains the leading cause of cancer-related mortality worldwide, with the phenomenon of Spread Through Air Spaces (STAS) emerging as a critical prognostic determinant since its recognition in the 2015 World Health Organization (WHO) classification (Kadota et al., 2015; Travis et al., 2015; Sung et al., 2021; Orlandi et al., 2025). Defined as the migration of tumor cells into air spaces beyond the main tumor edge, STAS is an independent risk factor for locoregional recurrence and reduced overall survival, particularly in patients with early-stage disease (Yang et al., 2021). Consequently, the presence of STAS has profound implications for surgical decision-making, as STAS-positive patients often benefit from lobectomy rather than sublobar resection to mitigate recurrence risks (Eguchi et al., 2019). However, the preoperative and intraoperative diagnosis of STAS remains a significant clinical challenge, and recent studies have explored pretreatment and intraoperative approaches for STAS prediction and frozen-section assessment. Frozen section analysis is commonly used for intraoperative assessment but is limited by imperfect sensitivity and susceptibility to artifacts (Walts and Marchevsky, 2018; Villalba et al., 2021; Ding et al., 2022; Ding et al., 2023). Recent multi-omics studies suggest that the intratumoral microbiome may be associated with tumor progression, metabolic reprogramming, and immune microenvironment modulation in lung cancer, but its relationship with the STAS phenotype remains unclear (Ma et al., 2024; Shi et al., 2024). In this study, we integrated microbiome and metabolomic analyses to investigate whether Corynebacterium enrichment is associated with phospholipid metabolic alterations and STAS, and to explore a possible link with ABCA3-related lipid homeostasis.

Historically considered a sterile organ, the lung is now recognized to harbor a diverse and dynamic microbiome that undergoes significant dysbiosis during tumorigenesis (Dickson and Huffnagle, 2015; Nejman et al., 2020). Emerging evidence utilizing next-generation sequencing suggests that the intratumoral microbiome is not merely a passenger but an active component of the tumor microenvironment (TME) capable of modulating cancer progression, metastasis, and host immune responses (Shi et al., 2024). For instance, specific intratumoral taxa have been shown to promote lung cancer metastasis through metabolite-mediated epigenetic remodeling or to influence therapeutic efficacy by altering immune cell infiltration (Ma et al., 2024). While genera such as Streptococcus, Veillonella, and Corynebacterium have been identified as prevalent constituents of the lung cancer microbiota, their specific mechanistic contributions to distinct invasive phenotypes remain largely unexplored (Li et al., 2023; Shi et al., 2024). In particular, it is currently unknown whether the enrichment of Corynebacterium contributes to the metabolic reprogramming required for the unique aerogenous dissemination observed in STAS-positive adenocarcinomas.

Metabolic reprogramming constitutes a hallmark of cancer (Hanahan, 2022). It enables malignant cells to adapt to the nutrient-poor tumor microenvironment. This adaptation supports unrestricted proliferation and invasion. In lung adenocarcinoma, alterations in lipid metabolism are particularly critical for tumor progression and metastasis (Faubert et al., 2020; Bian et al., 2025). These alterations specifically involve glycerophospholipid synthesis and membrane remodeling. Emerging evidence suggests that the intratumoral microbiome is a potent regulator of this metabolic rewiring. Intratumoral bacteria can modulate cancer cell metabolism either by depleting nutrients or by secreting bioactive metabolites. For instance, a recent study demonstrated that butyrate derived from the intratumoral microbiome promotes lung cancer metastasis. This occurs through the modulation of gene expression and immune responses (Ma et al., 2024). Despite these advances, the specific metabolic crosstalk driving the unique STAS phenotype is still poorly researched. Corynebacterium has been identified as a prevalent genus in the lung microbiota (Dickson and Huffnagle, 2015; Yang et al., 2024). However, it is currently unknown whether its enrichment alters the lipidomic landscape mediated by transporters such as ABCA3. These changes could allow tumor cells to detach and spread through air spaces.

To address these questions, we integrated 16S rRNA gene sequencing and untargeted metabolomics to characterize the STAS-associated microenvironment. We observed higher Corynebacterium prevalence and relative abundance in tumor tissues and correlations between Corynebacterium and glycerophospholipid-related metabolites, which supported the development of an exploratory microbial-metabolic signature for STAS risk stratification. We further used public transcriptomic data to explore whether ABCA3 and MMP9, genes related to lipid homeostasis and invasion, may provide external biological context for these associations. We also performed qRT-PCR in selected same-cohort tumor samples to evaluate ABCA3 and MMP9 expression in the same tissue context.

## 2 Materials and methods

### 2.1 Study population and sample collection

This retrospective study enrolled patients with pathologically confirmed lung adenocarcinoma (LUAD) who underwent curative-intent surgical resection (lobectomy or sublobar resection) at The Affiliated Wuxi People’s Hospital of Nanjing Medical University between 2024 and 2025. To reduce potential confounding effects on the intratumoral microbiome and metabolome, patients were excluded if they had received neoadjuvant chemotherapy and/or radiotherapy, or systemic antibiotic therapy within 3 months prior to surgery. Relevant clinicopathological variables were collected from medical records. The study was conducted in accordance with the Declaration of Helsinki and approved by the Institutional Review Board of The Affiliated Wuxi People’s Hospital of Nanjing Medical University (Approval No. KY24150). Written informed consent was obtained in accordance with institutional policies.

Spread through air spaces (STAS) status was determined on hematoxylin–eosin-stained sections according to the 2015 WHO-recognized definition (tumor cell clusters, micropapillary structures, or single tumor cells within air spaces beyond the main tumor edge). STAS was assessed by two experienced thoracic pathologists blinded to multi-omics results, and discrepant cases were resolved by consensus review.

Fresh tumor tissues and paired adjacent non-tumor lung tissues were collected intraoperatively using sterile instruments to minimize environmental contamination, immediately snap-frozen in liquid nitrogen, and stored at -80 °C until downstream analyses. Given the low microbial biomass of lung tissue samples, contamination control was incorporated at multiple steps. Sampling controls (n=5), DNA extraction blanks (n=5), and no-template PCR controls (n=5) were included to monitor contamination introduced during tissue handling, DNA extraction, and PCR amplification, respectively (Salter et al., 2014). Candidate contaminants were identified by comparing taxa detected in negative controls with those detected in tissue samples. Taxa showing evidence of background or reagent contamination were excluded from downstream analyses, and the remaining taxa were used for beta-diversity, differential abundance, and correlation analyses. For microbiome profiling, 19 paired tumor (T) and adjacent normal (N) tissues were analyzed (38 tissues total), consistent with the 5R 16S sequencing report.

### 2.2 16S rRNA gene sequencing and microbiome analysis

16S rRNA gene sequencing and primary bioinformatics were performed by Shanghai Biotree Biotech Co., Ltd. (Shanghai, China) using 5R 16S rRNA sequencing, which amplifies five regions (R1–R5) of the bacterial 16S rRNA gene via multiplex PCR to increase taxonomic resolution in low-biomass tissues. Sequencing reads from the five regions were integrated and taxonomically assigned using the Short MUltiple Regions Framework (SMURF), which reconstructs the most likely 16S sequence composition across regions using an expectation–maximization algorithm, with an optimized Greengenes database (May 2013 version) for taxonomy annotation. To reduce low-abundance noise, read counts were normalized per sample and samples with total reads <1,000 (including negative controls) were removed; taxa with relative abundance <1×10–4 were filtered. Potential contaminants were identified by comparing taxa detected in tissue samples with those present in sampling controls, DNA extraction blanks, and no-template PCR controls, using a prevalence threshold of 30% in negative controls. Candidate contaminant taxa were removed from experimental samples before downstream beta-diversity, differential abundance, and correlation analyses (Salter et al., 2014; Davis et al., 2018). Beta-diversity was computed using Bray–Curtis distances and visualized by principal coordinates analysis (PCoA), with group differences tested using ANOSIM/PermANOVA as appropriate. Differentially abundant taxa were identified using LEfSe with P < 0.05 and an LDA score >3.0, and the relative abundance of Corynebacterium was further quantified between groups.

### 2.3 Untargeted metabolomics analysis

Untargeted metabolomics, including data acquisition and initial data processing, was performed by Shanghai Biotree Biotech Co., Ltd. (Shanghai, China) using an UHPLC–MS/MS platform consisting of an UHPLC system coupled to a quadrupole–Orbitrap high-resolution mass spectrometer (Thermo Fisher Scientific, USA). The metabolomics cohort was stratified according to the number of pathological high-risk features, including STAS, pleural involvement, and vascular tumor thrombus. Group 0 included tumors with no high-risk feature, Group 1 included tumors with one high-risk feature, and Group 2 included tumors with two or more high-risk features. The metabolomics dataset comprised 16 tumor tissues and 18 adjacent-normal tissues, together with five pooled quality-control (QC) samples. The STAS-specific metabolomic analysis included the 16 tumor tissues, comprising 8 STAS-positive and 8 STAS-negative tumors. Raw peak tables were subjected to a relative standard deviation (RSD/CV)-based de-noising filter. Features were retained when missing values did not exceed 50% within any group or across all groups; remaining missing values were imputed with half of the minimum value. Total ion current (TIC) normalization was applied prior to statistical modeling. The normalized data were imported into SIMCA (v18.0.1; Sartorius Stedim Data Analytics AB, Umeå, Sweden) for multivariate analysis. Principal component analysis (PCA) was performed after logarithmic transformation and centering (CTR), and outliers were assessed using the Hotelling’s T-squared 95% confidence ellipse. Orthogonal projections to latent structures–discriminant analysis (OPLS-DA) was conducted after logarithmic transformation and unit-variance (UV) scaling; model quality was evaluated by 7-fold cross-validation and 200 permutation tests. Differential metabolites were defined as those with VIP>1 in OPLS-DA and P < 0.05 in Student’s t-test, and were visualized using volcano plots. Pathway enrichment and topology analyses were performed using KEGG and MetaboAnalyst.

### 2.4 Integrative multi-omics and predictive modeling

Spearman correlation analysis was used to evaluate associations between selected bacterial taxa and differential metabolites. Correlation matrices were visualized using heatmaps and scatter plots. Correlation networks of glycerophospholipids and other lipid-like molecules were constructed based on significant pairwise correlations and visualized using R packages including corrplot (v0.89), network (v1.16.1), igraph (v1.2.6) and ggraph (v2.0.5). For diagnostic evaluation, receiver operating characteristic (ROC) curves and area under the curve (AUC) values were calculated using the R packages plotROC (v2.2.1) and pROC (v1.16.2). An integrated model combining Corynebacterium abundance, PC(39:6), and SM(d18:1/17:0) was constructed, and model performance was evaluated by AUC. The combined model was used to explore whether microbial and metabolic features could discriminate STAS status in the discovery cohort. Because of the small sample size and lack of an independent validation cohort, the model was considered exploratory.

### 2.5 Transcriptomic and clinical survival analysis

**Figure 1:**
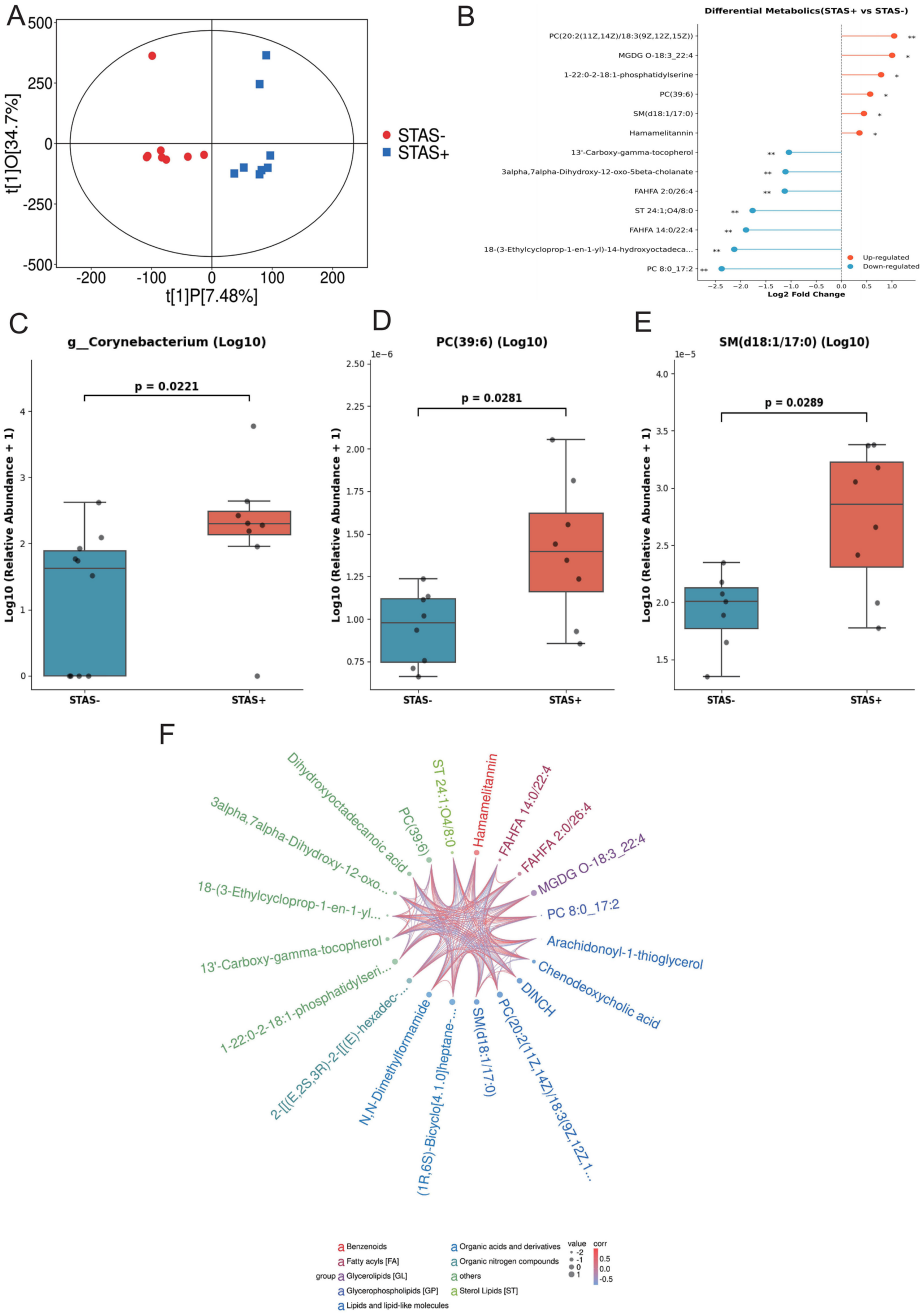
Intratumoral Corynebacterium and phospholipid metabolic features associated with STAS in LUAD. **(A)** OPLS-DA score plot showing metabolic differences between STAS-positive (STAS+,n=8) and STAS-negative (STAS−,n = 8) tumors. **(B)** Lollipop plot of selected differential metabolites between the STAS+ and STAS− groups. **(C)** Relative abundance of Corynebacterium in STAS+ and STAS− tumors. **(D, E)** Representative phosphatidylcholine (PC) and sphingomyelin (SM) species increased in STAS+ tumors. **(F)** Correlation network showing associations among glycerophospholipids and other lipid-like molecules in STAS+ tumors.

For same-cohort expression validation, qRT-PCR was performed in selected tumor samples from the discovery cohort, including six STAS-positive/Corynebacterium-enriched tumors and six STAS-negative/Corynebacterium-low tumors. This targeted subset was selected according to concordant STAS status and Corynebacterium abundance and was separate from the complete STAS-specific metabolomic cohort shown in **Figure 1A**. β-tubulin was used as the internal reference gene. Relative expression was calculated using the 2^-ΔCt method, and between-group differences were assessed using the Mann-Whitney U test.

To evaluate the clinical relevance of the lipid transporter ABCA3, its expression correlation and prognostic value was analyzed using the GEPIA2 (Gene Expression Profiling Interactive Analysis, version 2) database (http://gepia2.cancer-pku.cn/), which is based on TCGA (The Cancer Genome Atlas) and GTEx data comprising 483 tumor tissues and 59 normal lung tissues. The correlation between ABCA3 and MMP9 expression in LUAD was evaluated using Pearson’s correlation coefficients on log2-transformed transcripts per million (TPM) values. For survival analysis, LUAD patients were stratified into high- and low-expression groups based on the median expression level of ABCA3. Overall survival (OS) was visualized via Kaplan-Meier curves, with hazard ratios (HR) and 95% confidence intervals calculated through the Cox proportional hazards model, and the log-rank test was employed to assess statistical significance (p < 0.05).

### 2.6 Statistical analysis

Unless otherwise specified, statistical analyses were performed in R (base v4.5.2). Continuous variables were compared using parametric or non-parametric tests as appropriate. Correlations were assessed using Spearman’s rank correlation. Differences in Corynebacterium detection frequency were assessed using a two-sided Fisher’s exact test and, because the tumor and adjacent-normal tissues were paired within patients, an exact McNemar test. All tests were two-sided, and P < 0.05 was considered statistically significant.

## 3 Results

### 3.1 Distinct intratumoral microbiome signature in lung adenocarcinoma characterized by Corynebacterium enrichment

**Figure 2:**
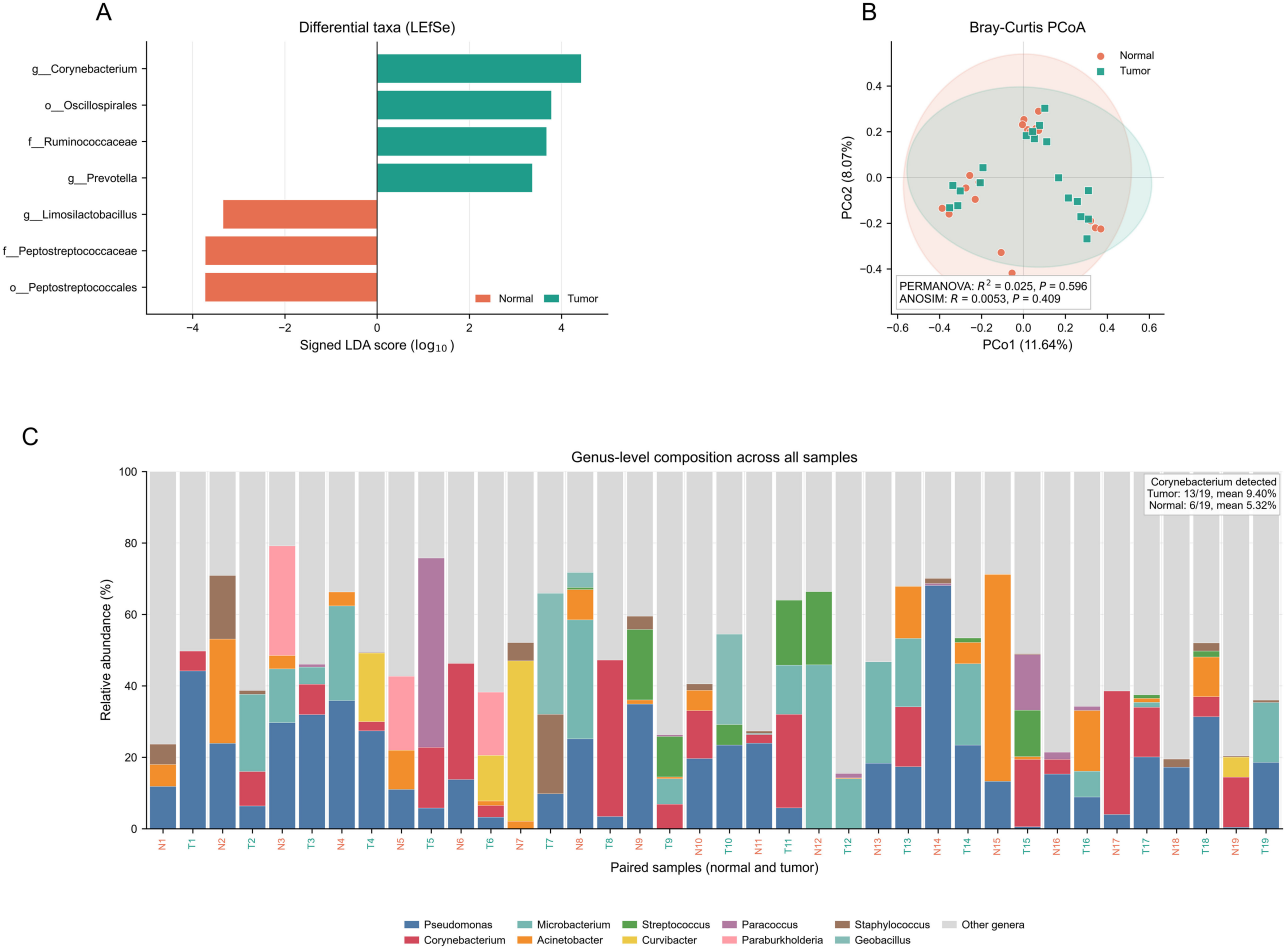
Taxon-level microbiome differences in lung adenocarcinoma tissues. **(A)** LEfSe analysis identifying differentially abundant taxa between tumor and adjacent normal tissues. Corynebacterium was identified as a tumor-enriched genus-level feature (LDA score > 4.0, P < 0.05). **(B)** Principal Coordinate Analysis (PCoA) based on Bray-Curtis distance showing partial visual dispersion between tumor (T) and adjacent normal (N) tissues; global community differences were not statistically significant by PERMANOVA/adonis or ANOSIM. **(C)** Relative abundance of top bacterial genera across all samples.

To investigate the microbial landscape of lung adenocarcinoma (LUAD), we compared the community structures of tumor (T) and adjacent normal (N) tissues. Principal Coordinate Analysis (PCoA) based on Bray-Curtis distances showed partial visual dispersion between tumor and adjacent normal tissues, but the global community difference was not statistically significant by PERMANOVA/adonis (R2 = 0.025, P = 0.596) or ANOSIM (R = 0.0053, P = 0.409; **Figure 2B**).

Because global community tests and individual-taxon analyses address distinct hypotheses, we next performed an exploratory LEfSe analysis to determine whether specific taxa showed localized group-associated patterns. This analysis identified Corynebacterium as the strongest tumor-associated taxon-level signal (LDA score > 4.0, P < 0.05; **Figure 2A**). Other tumor-associated taxa included Oscillospirales and Prevotella, while the normal group was characterized by Limosilactobacillus. After contaminant screening based on sampling controls, DNA extraction blanks, and no-template PCR controls, Corynebacterium remained detectable in 13 of 19 tumor tissues and 6 of 19 adjacent normal tissues. However, the difference in detection frequency did not reach statistical significance by a two-sided Fisher’s exact test (odds ratio = 4.69, P = 0.0502) or by an exact McNemar test accounting for the paired sampling design (P = 0.0923). Its mean relative abundance was numerically higher in tumor tissues than in adjacent normal tissues (9.40% vs. 5.32%; **Figure 2C**), although this finding should be interpreted as a recurrent taxon-level signal rather than definitive evidence of viable intratumoral colonization. These data suggest a recurrent taxon-level Corynebacterium-associated signal in a subset of LUAD tissues, but do not establish a broad global microbiome shift or prove viable intratumoral colonization.

### 3.2 LUAD displays distinct metabolic profiles with prominent phospholipid metabolic reprogramming

**Figure 3:**
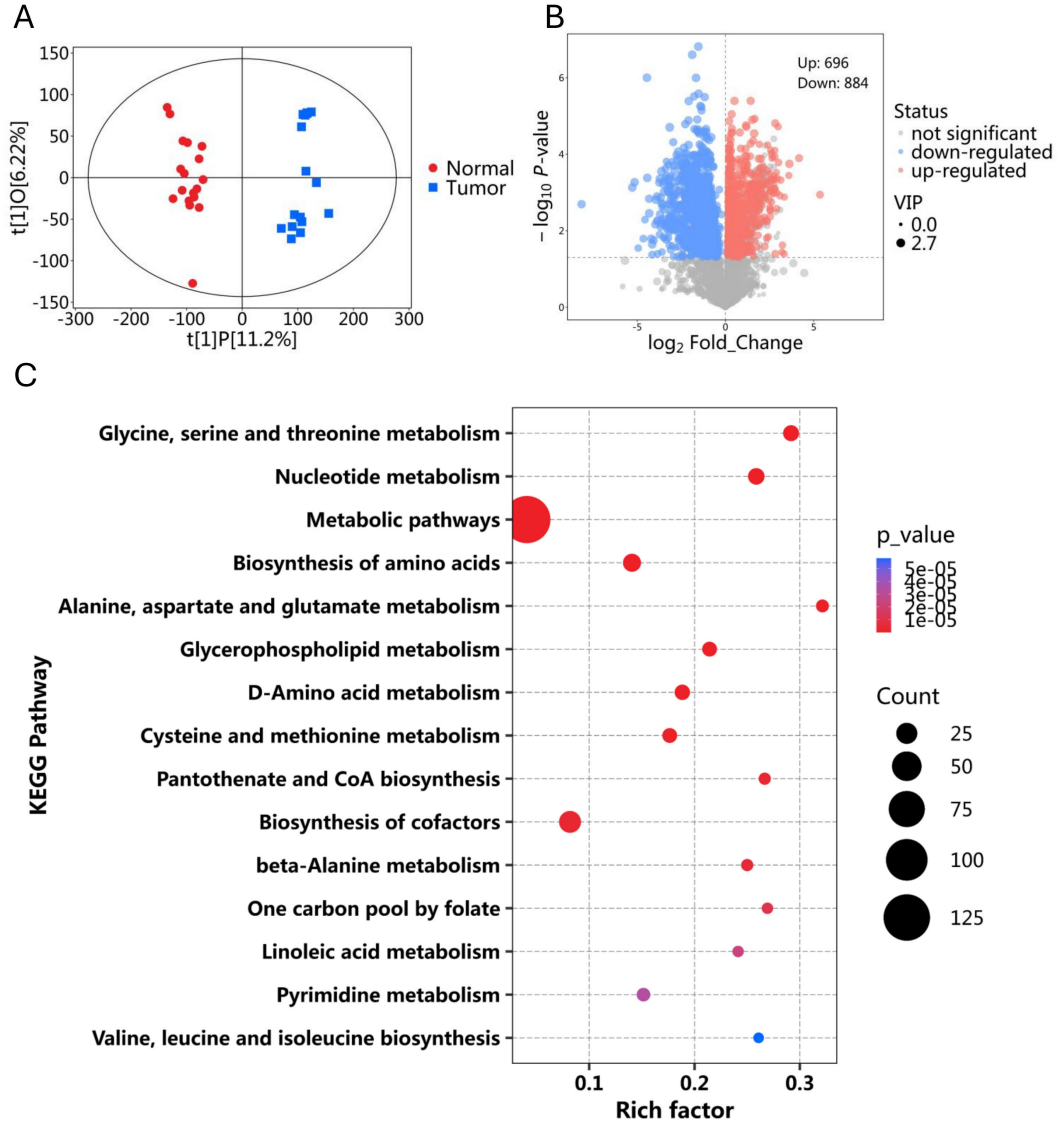
Metabolomic profiling and pathway enrichment analysis in LUAD tissues. **(A)** OPLS-DA score plot showing metabolic differences between adjacent normal (red) and tumor (blue) tissues. **(B)** Volcano plot of differentially abundant metabolites; red dots represent 696 up-regulated metabolites, while blue dots represent 884 down-regulated metabolites in tumor tissues (P < 0.05). **(C)** KEGG pathway enrichment bubble plot showing altered metabolic pathways, including glycerophospholipid metabolism.

To further elucidate the functional changes within the LUAD microenvironment, we conducted untargeted metabolomic profiling on paired tumor and normal tissues. Orthogonal Projections to Latent Structures–Discriminant Analysis (OPLS-DA) revealed a clear and robust separation between the metabolic signatures of the tumor and normal groups (**Figure 3A**). This distinct clustering indicates that the malignancy is accompanied by a systematic shift in the global metabolic landscape, reflecting the unique physiological requirements of tumor cells.

Quantitative analysis of the metabolic features was visualized through a volcano plot, which identified a total of 1,580 significantly altered metabolites. Specifically, 696 metabolites were up-regulated and 884 were down-regulated in the tumor tissues compared to adjacent normal tissues (**Figure 3B**). This broad dysregulation underscores the complexity of metabolic adaptive mechanisms in LUAD.

To identify the biological significance of these changes, we performed KEGG pathway enrichment analysis. The results highlighted significant alterations in lipid and amino acid metabolism, with Glycerophospholipid metabolism and Linoleic acid metabolism being among the most prominently enriched pathways (**Figure 3C**). Additionally, pathways such as glycine, serine, and threonine metabolism, as well as alanine, aspartate, and glutamate metabolism, showed high rich factors and significant P-values. These findings support the presence of phospholipid metabolic reprogramming in LUAD and provide a basis for evaluating its association with Corynebacterium abundance.

### 3.3 Intratumoral Corynebacterium taxa are associated with membrane lipid accumulation

**Figure 4:**
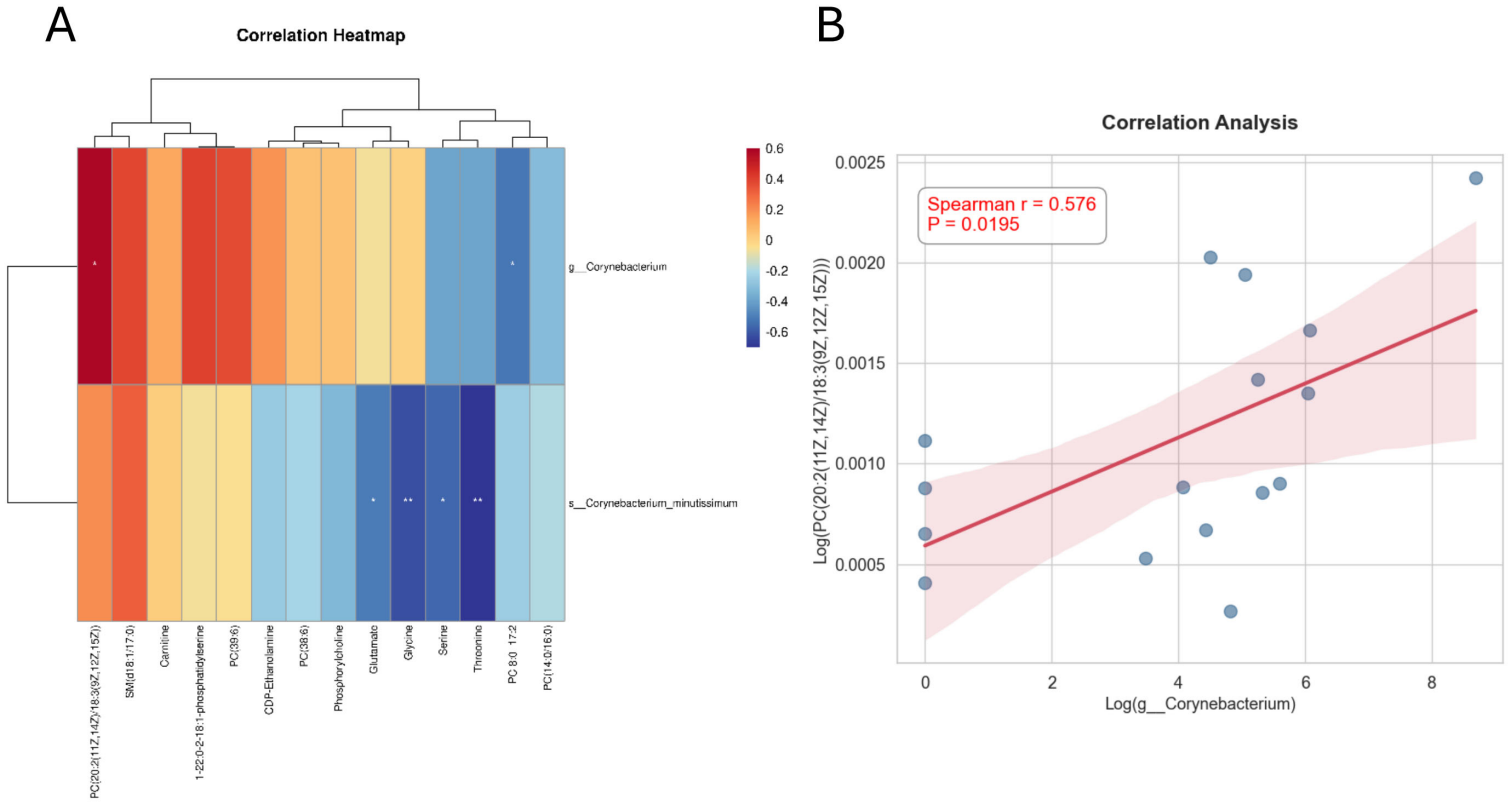
Specific Corynebacterium taxa are associated with amino-acid-related and lipid-related metabolic differences. **(A)** Heatmap showing Spearman correlations between bacterial abundance (rows) and differential metabolites (columns). Blue clusters indicate a significant negative correlation between amino acids and **(C)** minutissimum (nutrient depletion); red clusters indicate positive correlations between membrane lipids/precursors and the genus Corynebacterium (lipid remodeling). **(B)** Scatter plot illustrating the positive correlation between Corynebacterium and phosphatidylcholine (PC) levels (r = 0.576, P = 0.0195).Colors indicate Spearman correlation coefficients; *P < 0.05 and **P < 0.01.

To investigate the potential functional relationship between the enriched microbiome and the altered metabolome, we performed Spearman correlation analysis between specific bacterial taxa and differentially abundant metabolites. The resulting heatmap revealed a distinct metabolic signature associated with Corynebacterium species (**Figure 4A**). Specifically, key amino acids, including Glutamate, Serine, Glycine, and Threonine, exhibited significant negative correlations with C. minutissimum, suggesting a localized depletion of these nutrients within the tumor microenvironment (**Figure 4A**).

In contrast, the genus Corynebacterium and the species C. minutissimum were significantly positively correlated with membrane lipids, such as phosphatidylcholine (PC) and sphingomyelin (SM), as well as the precursor CDP-Ethanolamine (**Figure 4A**). This relationship was further substantiated by quantitative analysis, which showed a robust positive correlation between Corynebacterium abundance and PC levels (r = 0.576, P = 0.0195; **Figure 4B**). Together, these findings suggest that Corynebacterium abundance is associated with membrane-lipid accumulation and amino-acid-related metabolic differences in LUAD tissues.

### 3.4 Intratumoral Corynebacterium and phospholipid metabolic reprogramming are associated with STAS in LUAD

To evaluate the clinical relevance of the microbial-metabolic axis, we investigated whether the enrichment of Corynebacterium and its associated metabolic shifts could predict the presence of STAS. OPLS-DA revealed a distinct metabolic separation between STAS-positive (STAS+) and STAS-negative (STAS-) tumors, suggesting that the presence of STAS is accompanied by a unique intratumoral chemical microenvironment (**Figure 1A**).

Quantitative analysis of differential metabolites showed a significant accumulation of membrane lipids in STAS+ tissues. Specifically, Phosphatidylcholine (PC) and Sphingomyelin (SM) species were significantly up-regulated in tumors exhibiting STAS (**Figures 1B, D, E**). Conversely, metabolites such as FAHFA and ST were significantly down-regulated (**Figure 1B**). Crucially, the relative abundance of the genus Corynebacterium was significantly higher in the STAS+ group compared to the STAS- group (P = 0.0221; **Figure 1C**).

Furthermore, correlation network analysis (**Figure 1F**) highlighted a dense cluster of interactions among glycerophospholipids (GP) and other lipid-like molecules in STAS+ tumors. These findings suggest that higher intratumoral Corynebacterium abundance is associated with a phospholipid-rich metabolic state in STAS-positive LUAD, supporting its evaluation as an exploratory marker of this invasive phenotype.

### 3.5 Exploratory microbial-metabolic signature for STAS discrimination

To evaluate the clinical diagnostic potential of the identified microbial and metabolic markers, we performed Receiver Operating Characteristic (ROC) curve analysis to determine their ability to predict the presence of Spread Through Air Spaces (STAS). Individual markers, including the abundance of Corynebacterium (AUC = 0.83), PC(39:6) (AUC = 0.83), and SM(d18:1/17:0) (AUC = 0.81), each showed apparent discriminatory ability for STAS status in this cohort.

**Figure 5:**
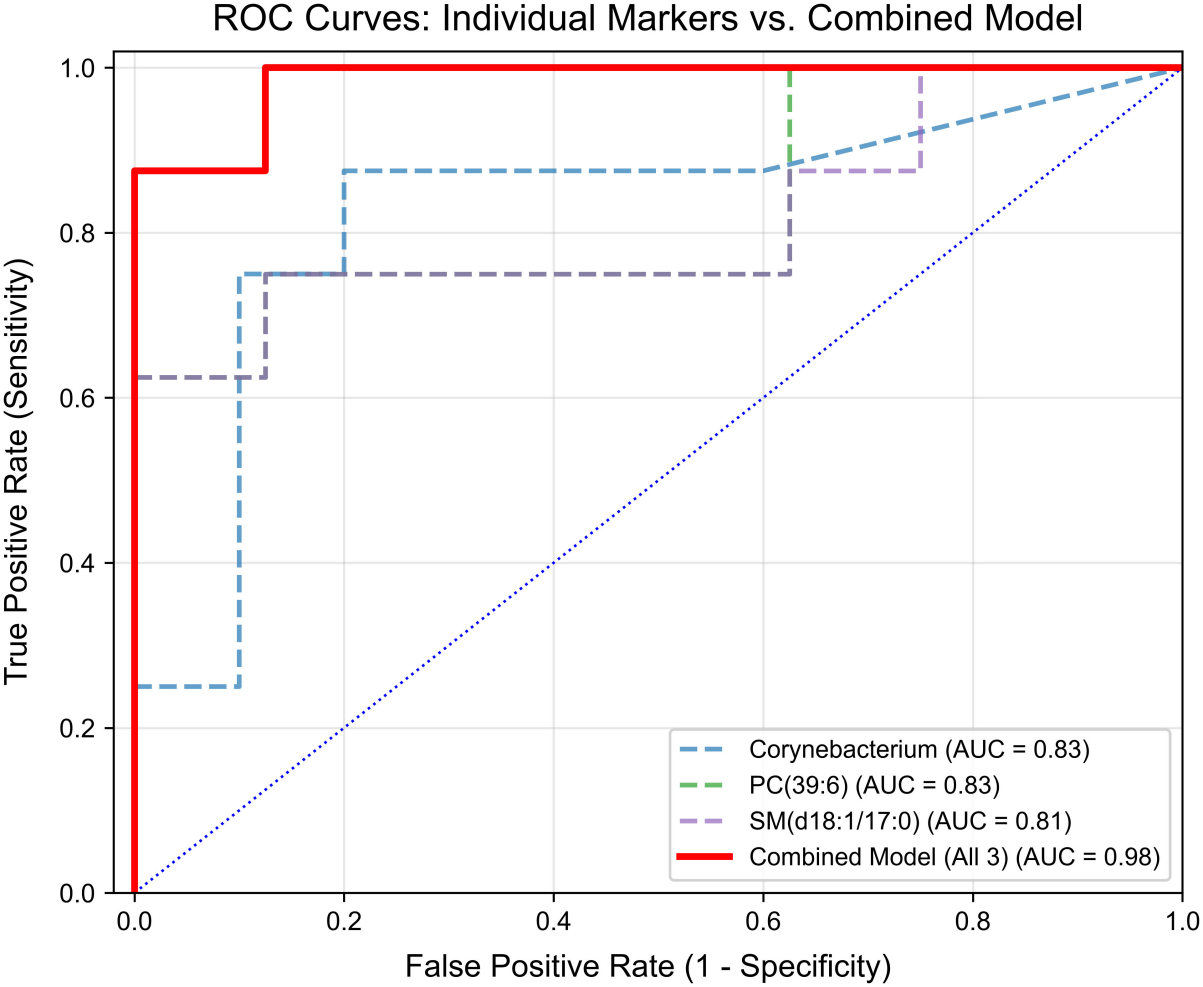
Exploratory microbial-metabolic signature for STAS discrimination. Receiver operating characteristic (ROC) curves showing the apparent performance of Corynebacterium abundance, PC(39:6), SM(d18:1/17:0), and the combined three-marker model for discriminating STAS-positive from STAS-negative LUAD in the discovery cohort.

Crucially, we developed an integrated model by combining these three microbial and metabolic features. The combined model achieved a high apparent AUC of 0.98 in the discovery dataset (**Figure 5**). Given the small sample size and absence of an independent validation cohort, this result should be interpreted as exploratory and potentially susceptible to overfitting. This synergistic effect suggests that the crosstalk between intratumoral Corynebacterium and the surrounding lipid microenvironment provides a highly sensitive and specific biological signature for identifying aggressive LUAD phenotypes prone to airway dissemination.

### 3.6 Exploratory transcriptomic analysis of ABCA3 and MMP9 in LUAD

In selected tumor samples from the discovery cohort, qRT-PCR analysis showed that the STAS-positive/Corynebacterium-enriched group had significantly lower ABCA3 expression than the STAS-negative/Corynebacterium-low group (median relative expression: 0.57 vs. 2.02; P = 0<0.0001). Conversely, MMP9 expression was significantly higher in the STAS-positive/Corynebacterium-enriched group (median relative expression: 1.79 vs. 0.87; P = 0.0011). ABCA3 and MMP9 expression were negatively correlated across the 12 analyzed samples (Spearman rho = -0.699, P = 0.011).

**Figure 6:**
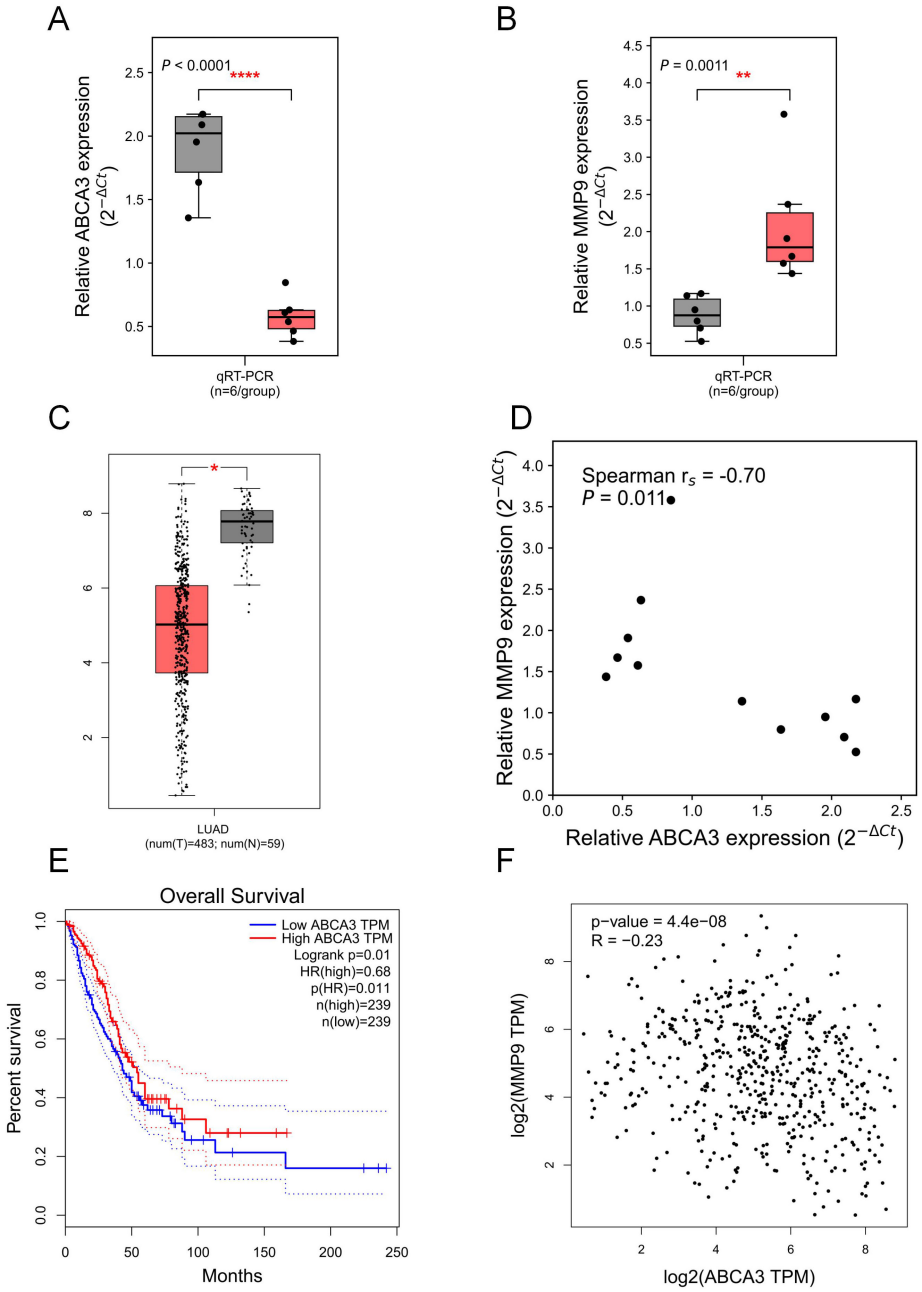
qRT-PCR validation and exploratory transcriptomic analysis of ABCA3 and MMP9 in LUAD. **(A)** qRT-PCR analysis showing lower ABCA3 expression in the STAS-positive/Corynebacterium-enriched group than in the STAS-negative/Corynebacterium-low group (n = 6 per group). **(B)** qRT-PCR analysis showing higher MMP9 expression in the STAS-positive/Corynebacterium-enriched group(n = 6 per group). **(C)** Public transcriptomic analysis of ABCA3 mRNA expression in LUAD tumor tissues (T, n = 483) and normal lung tissues (N, n = 59). **(D)** Correlation between ABCA3 and MMP9 expression across the 12 qRT-PCR-analyzed tumor samples(n=12). **(E)** Kaplan-Meier survival curves showing overall survival according to high versus low ABCA3 expression. **(F)** Public transcriptomic correlation between ABCA3 and MMP9 expression in LUAD. These data provide same-sample expression-level support and external biological context, but do not directly establish an ABCA3-MMP9-STAS mechanism.

To evaluate the clinical relevance of the identified metabolic shifts, we investigated the expression and prognostic significance of ABCA3 (ATP-binding cassette transporter A3), a key transporter involved in lung phospholipid homeostasis. Analysis of the LUAD cohort revealed distinct mRNA expression levels of ABCA3 across 483 tumor (T) and 59 normal (N) tissue samples (**Figure 6A**).

Kaplan-Meier survival analysis (n=478) demonstrated that patients with high ABCA3 expression levels had significantly better overall survival (OS) compared to those with low expression (HR = 0.68, P = 0.01; **Figure 6B**). Furthermore, correlation analysis showed that ABCA3 expression was significantly and negatively correlated with MMP9 (matrix metallopeptidase 9), a critical marker of tumor invasion and extracellular matrix degradation (R = −0.23, P = 4.4 × 10−8; **Figure 6C**). Because public transcriptomic datasets do not include STAS annotation and ABCA3/MMP9 were not measured in the same tissue samples used for microbiome-metabolome profiling, these findings should be interpreted as exploratory biological context rather than direct evidence of an ABCA3-MMP9-STAS mechanism. Together with the same-cohort qRT-PCR results, these data provide expression-level support for an ABCA3-low/MMP9-high invasive pattern, but they do not establish a causal ABCA3-MMP9-STAS mechanism.

## 4 Discussion

The accurate identification of spread through air spaces is essential for guiding surgical resection in lung adenocarcinoma as it significantly correlates with poor prognosis and locoregional recurrence (Kadota et al., 2012; Eguchi et al., 2019). Addressing the current diagnostic limitations, this study utilized a multi-omics approach to characterize the specific microenvironment associated with this invasive pattern, complementing recent efforts that used pretreatment prediction, frozen-section assessment, and single-cell/spatial transcriptomic approaches to evaluate STAS biology and diagnosis (Ding et al., 2022; Ding et al., 2023; Ding et al., 2025). We discovered a robust association between Corynebacterium abundance and glycerophospholipid accumulation, which served as the basis for a highly accurate diagnostic model yielding an AUC of 0.98. In addition, public transcriptomic analyses provided external context suggesting that ABCA3-related lipid homeostasis and MMP9-related invasion programs may be clinically relevant in LUAD, although these analyses do not establish a direct STAS-specific mechanism.

While the intratumoral microbiome is increasingly recognized as a functional component of the tumor microenvironment (TME), its specific contribution to distinct invasion patterns has remained elusive (Nejman et al., 2020; Cao et al., 2024). Our study identified a significant enrichment of Corynebacterium in STAS-positive tissues, distinguishing them from STAS-negative counterparts. Although Corynebacterium is a common commensal of the upper respiratory tract and has been previously detected in lung cancer tissues, its specific association with the STAS phenotype represents a novel finding (Yang et al., 2024). From a biological perspective, members of the genus Corynebacterium have lipid-rich cell envelope features, and some species, such as the lipophilic C. kroppenstedtii, show genomic and physiological adaptations related to lipid-rich environments (Tauch et al., 2008; Modak et al., 2025). These observations provide background plausibility for a microbiome-lipid association, but they do not establish that Corynebacterium directly induces phospholipid remodeling in LUAD

Previous studies have primarily linked genera such as Streptococcus, Veillonella, or Prevotella to lung carcinogenesis or immune modulation (Shi et al., 2024). For instance, Streptococcus has been shown to upregulate the PI3K/Akt pathway to promote cell proliferation, while intratumoral Escherichia has been associated with improved responses to immune checkpoint inhibitors (Tsay et al., 2021; Elkrief et al., 2024). In this context, our data suggest that Corynebacterium may mark a microbial niche associated with STAS-positive tumors. Whether this association reflects bacterial adaptation to an altered tumor microenvironment or a functional contribution to invasion requires experimental validation.

The accumulation of glycerophospholipids in tumors exhibiting spread through air spaces indicates metabolic reprogramming within the tumor microenvironment (Bian et al., 2025). This lipidomic alteration was positively correlated with Corynebacterium abundance, suggesting a microbiome-metabolome association involving membrane lipid metabolism. Given the lipid-related biology described in corynebacteria, this association is biologically plausible, but the present data remain correlative and require functional validation (Tauch et al., 2008; Modak et al., 2025).This interpretation is consistent with recent evidence that intratumoral microbiota can be linked to lung cancer metastasis through bioactive metabolites and host gene-expression changes (Ma et al., 2024).In our study, public transcriptomic analyses showed that ABCA3 expression was associated with LUAD prognosis and inversely correlated with MMP9 expression. Given the critical role of ABCA3 in maintaining pulmonary surfactant homeostasis, its downregulation likely disrupts cell adhesion mechanics (Matsuzaki et al., 2008). This loss of function was further associated with the upregulation of matrix metalloproteinase-9, an enzyme known to degrade the extracellular matrix and facilitate the detachment of tumor cells into alveolar spaces (Orlandi et al., 2025). These data collectively elucidate a potential signaling axis connecting the intratumoral microbiome and lipid metabolism to the invasive phenotype of spread through air spaces. In selected same-cohort tumor samples, qRT-PCR further showed lower ABCA3 and higher MMP9 expression in STAS-positive/Corynebacterium-enriched tumors. These expression findings support an ABCA3-low/MMP9-high invasive phenotype associated with STAS-positive tumors, but do not demonstrate direct regulation by Corynebacterium.

The clinical translation of these findings holds significant promise for refining surgical decision-making in early-stage lung adenocarcinoma (Orlandi et al., 2025). Accurate preoperative and intraoperative diagnosis of spread through air spaces remains a challenge, despite recent efforts to develop pretreatment predictive models and evaluate frozen-section diagnosis, because intraoperative assessment still has imperfect sensitivity and may be affected by artifacts such as spread through a knife surface (Walts and Marchevsky, 2018; Ding et al., 2022; Ding et al., 2023). Consequently, surgeons often face the dilemma of choosing between sublobar resection and lobectomy without definitive pathological guidance (Weiss and Rochefort, 2019). Our multi-omics model achieved a high apparent AUC of 0.98 in the discovery cohort, suggesting that microbial-metabolic features may have exploratory value as ancillary risk-stratification markers, but the model requires independent validation before clinical application. Given that patients with spread through air spaces exhibit significantly higher recurrence rates following limited resection compared to lobectomy, the preoperative or intraoperative identification of the Corynebacterium and glycerophospholipid signature could identify high-risk candidates who would benefit from anatomical lobectomy (Yang et al., 2021). Furthermore, establishing the microbial driver of this invasive phenotype raises the possibility of exploring antibiotic or metabolic interventions as neoadjuvant strategies to limit aerogenous dissemination prior to surgery (Cao et al., 2024).

Several limitations warrant consideration. The retrospective single-center design introduces potential selection bias that necessitates validation in larger multicenter cohorts to ensure generalizability. Additionally, lung tissue is a low-microbial-biomass specimen type. Although sampling controls, DNA extraction blanks, and no-template PCR controls were included and contaminant filtering was performed, residual background contamination cannot be completely excluded, particularly for genera that may overlap with reagent or sampling contaminants (Salter et al., 2014; Davis et al., 2018). Therefore, Corynebacterium should be interpreted as a recurrent taxon-level signal associated with LUAD tissues rather than definitive evidence of viable intratumoral colonization. Finally, the microbiome, metabolome, and public transcriptomic analyses are correlative. Functional *in vitro* and *in vivo* studies are required to determine whether Corynebacterium has a causal role in lipid metabolic remodeling or STAS biology. Although qRT-PCR provided same-sample support for differential ABCA3 and MMP9 expression, the small validation subset and observational design preclude causal inference.

## 5 Conclusions

In conclusion, this study suggests that intratumoral Corynebacterium enrichment and glycerophospholipid accumulation are associated features of LUAD exhibiting STAS. These microbial and metabolic alterations may define an exploratory STAS-associated signature and provide a basis for future studies of microbiome-metabolome interactions in LUAD. Public transcriptomic analyses further suggest that ABCA3-related lipid homeostasis and MMP9-related invasion programs may be relevant to LUAD biology, but causal relationships and clinical utility require validation in larger independent cohorts and functional experimental models. Same-cohort qRT-PCR further supports the relevance of ABCA3-related lipid homeostasis and MMP9-related invasion programs in the selected samples.

## Data Availability

The original contributions presented in the study are included in the article/supplementary material. Further inquiries can be directed to the corresponding authors.
